# Natural Bioactive Peptides from Tree Peony Flowers: Multifunctional Effects on Skin Antioxidation, Wrinkle Reduction, Moisturization, and Melanin Inhibition

**DOI:** 10.3390/antiox15030350

**Published:** 2026-03-11

**Authors:** Yunzong Liu, Ruofei Zheng, Linyue Zhong, Junyang Huang, Xuefang Guan, Juqing Huang, Mei Xu, Yafeng Zheng, Qi Wang

**Affiliations:** 1 Institute of Food Science and Technology, Fujian Academy of Agricultural Sciences, Fuzhou 350003, China; liuyunzong0210@163.com (Y.L.); lingyue_zhong@163.com (L.Z.); guan-619@163.com (X.G.); jq_huang@zju.edu.cn (J.H.); 2 Yuanzhidao-FAAS Joint Innovation Center for Functional Probiotics & Medicinal-Edible Resources, Xiamen 361199, China; july@yzdbio.com (J.H.); 18259288898@163.com (M.X.); 3 Key Laboratory of Processing of Subtropical Special Fruits and Vegetables and Mushrooms, Ministry of Agriculture and Rural Affairs, Fuzhou 350002, China; 4 Fujian Key Laboratory of Agricultural Product (Food) Processing, Fuzhou 350002, China; 5 College of Food Science, Fujian Agriculture and Forestry University, Fuzhou 350002, China; zhengruofei2001@163.com (R.Z.); zyffst@163.com (Y.Z.)

**Keywords:** edible tree peony flower, protein hydrolysate, bioactive peptides, skin health, antioxidant, anti-wrinkle, whitening

## Abstract

The edible tree peony (*Paeonia suffruticosa* Andrews) flowers are rich in bioactive components with potential health benefits, but the skin-health-promoting effects of their protein hydrolysates remain understudied. The present research sought to evaluate the antioxidant, anti-wrinkle, moisturizing, and whitening properties of tree peony flower protein hydrolysate (TPFP). TPFP was prepared via enzymatic hydrolysis and ultrafiltration, and its peptide sequences were identified by liquid chromatography-tandem mass spectrometry (LC-MS/MS), revealing 54 unique small-molecule peptides with an average amino acid length of 8.2 residues and a molecular weight of 914.51 Da. In vitro safety evaluation using CCK-8 assay showed TPFP (20–100 μM) did not induce substantial cytotoxic effects in either HaCaT keratinocytes or B16F10 melanoma cell lines. Functional assays demonstrated that TPFP dose-dependently inhibited UVB-induced reactive oxygen species (ROS) overproduction and restored superoxide dismutase (SOD) and catalase (CAT) activities in HaCaT cells, exerting antioxidant effects. Additionally, TPFP protected pro-collagen I from UVB-induced loss, suppressed the expression of matrix metalloproteinase 1 (MMP-1), and restored hyaluronic acid (HA) content, showing anti-wrinkle and moisturizing potentials. In α-MSH-stimulated B16F10 cells, TPFP suppressed melanin synthesis by downregulating the protein expression of tyrosinase (TYR), tyrosinase-related protein 1 (TRP-1), and TRP-2, achieving a whitening effect. These findings indicate that TPFP possesses comprehensive skin-health-promoting activities with good biocompatibility, highlighting its potential as a natural functional ingredient in cosmetics and functional foods.

## 1. Introduction

Skin health is fundamental to overall well-being, serving as the body’s primary barrier against environmental stressors, pathogens, and moisture loss [1,2]. Beyond its protective function, skin appearance and vitality are closely linked to quality of life, with conditions such as photoaging, dryness, and hyperpigmentation posing significant aesthetic and psychological challenges for individuals worldwide [3]. Environmental factors like UVB irradiation, pollution, and oxidative stress are major drivers of skin damage: UVB exposure triggers abnormal overgeneration of reactive oxygen species (ROS), leading to collagen degradation, reduced hyaluronic acid (HA) content, and impaired antioxidant defense systems, ultimately resulting in wrinkles, dryness, and loss of elasticity [4,5]. Meanwhile, abnormal melanin accumulation, triggered by factors such as α-melanocyte-stimulating hormone (α-MSH), causes hyperpigmentation, mediated by key melanogenesis-related enzymes including tyrosinase (TYR), tyrosinase-related protein 1 (TRP-1), and TRP-2 [6]. Given these challenges, the development of natural, safe, and multifunctional ingredients for skin health promotion has become a critical focus in cosmetic and functional food research [7].

In recent years, bioactive peptides extracted from plants have become attractive options for various health applications, owing to their superior biocompatibility, low toxicity, and diverse physiological activities [8]. Plant protein hydrolysates, obtained via enzymatic hydrolysis, are rich in small-molecule peptides (typically 500–3000 Da) that can easily penetrate the skin barrier, be absorbed by cells, and exert targeted effects [9]. Significant advances have been made in this field: for instance, canary seed (*Phalaris canariensis* L.) peptides (CSPs) demonstrate dual inhibitory activity against elastase and tyrosinase, and favor skin permeation via key peptide molecular interactions, exerting remarkable anti-aging and skin-health-promoting effects for skincare applications [10]. Similarly, peptide fractions derived from the microalgae *Spirulina platensis* and *Chlorella vulgaris* through enzymatic processing display strong antioxidant and skin-aging enzyme inhibitory activities, exerting promising potential as viable, environmentally sustainable reservoirs of biofunctional peptides applicable across nutraceutical, therapeutic, and cosmeceutical sectors [11]. These findings highlight the versatility of plant protein peptides as multifunctional skin-health-promoting agents, driving growing interest in exploring new plant sources for peptide extraction.

*Paeonia suffruticosa* Andrews, commonly known as tree peony, holds significant cultural and medicinal value in China, with its flowers abundant in bioactive components such as proteins, phenolics, and flavonoids [12,13]. Among these, plant polyphenols exhibit prominent antioxidant properties, which endow them with considerable application potential in skin anti-aging by scavenging reactive oxygen species (ROS) [14]. Tree peony peptides have also been confirmed to possess antioxidant and cytoprotective activities in previous studies [12,13], yet research into the skin-health-promoting effects of tree peony flower protein hydrolysates (TPFPs) remains scarce. While bioactive peptides from various sources have been extensively studied for their skin-beneficial properties [15,16,17,18], notably, no natural occurrence of the currently widely used classic anti-aging peptides [19,20] (e.g., carnosine, copper tripeptide-1, Acetyl Tetrapeptide-2) has been reported in tree peony flowers to date. In particular, systematic studies on TPFP’s capacity to combat UVB-induced oxidative stress, as well as its potential anti-wrinkle, moisturizing and whitening activities, are still lacking. This critical research gap has restricted the comprehensive development and utilization of tree peony flowers as a natural resource with great potential for functional cosmetic and food ingredient production.

To address this limitation, the present study focused on TPFP prepared from edible tree peony flowers. Specifically, it aimed to first identify TPFP’s peptide sequences via liquid chromatography-tandem mass spectrometry (LC-MS/MS) and evaluate its in vitro safety through cytotoxicity assays on HaCaT keratinocytes and B16F10 melanoma cells. Second, it assessed TPFP’s antioxidant activity by measuring ROS scavenging and the restoration of SOD and CAT activities in UVB-irradiated HaCaT cells, as well as its anti-wrinkle and moisturizing effects by analyzing PCI, MMP-1, and HA levels. Third, it explored its whitening effect by determining melanin content and the expression of TYR, TRP-1, and TRP-2 in α-MSH-stimulated B16F10 cells. The findings aim to provide scientific evidence for developing TPFP as a natural multifunctional skin-health-promoting ingredient and expanding tree peony applications in cosmetics and functional foods.

## 2. Materials and Methods

### 2.1. Materials and Reagents

The dried flowers of the tree peony (*P*. *suffruticosa* Andrews) cultivar “Danfeng” were purchased from Shaanxi State-owned Peony Industry Group Co., Ltd. (Xian, China). Alkaline protease (enzyme activity > 200,000 U/g, optimal pH 8.5~10.5, optimal temperature 40~55 °C) was acquired from Solarbio Science & Technology (Beijing, China). Dulbecco’s modified Eagle medium (DMEM), fetal bovine serum (FBS), trypsin-EDTA (0.25%) and penicillin/streptomycin were supplied by Gibco (New York, NY, USA). 4′,6-diamidino-2-phenylindole (DAPI) was obtained from Sigma-Aldrich (St. Louis, MO, USA), and 2,7-dichlorodihydrofluorescein diacetate (DCFH-DA) was purchased from Beyotime (Shanghai, China). α-melanocyte stimulating hormone (α-MSH) was purchased from MedChemExpress LLC (Shanghai, China). The enzyme-linked immunosorbent assay (ELISA) kits of catalase (CAT) and total superoxide dismutase (T-SOD) were purchased from Elabscience Biotechnology (Wuhan, China). ELISA kits of human hyaluronic acid (HA), matrix metalloproteinase 1 (MMP-1) and procollagen I (PCI) were provided by ELK Biotechnology Co., Ltd. (Wuhan, China). Anti-TRP-1, anti-TRP-2, anti-tyrosinase and anti-β-actin antibodies were purchased from Abcam (Cambridge, UK).

### 2.2. Preparation of Protein Hydrolysate TPFP

This method was slightly modified based on previously reported protocols [21]. Fifty grams of dried tree peony flowers were crushed in a high-speed grinder (Model FW100, Tianjin, China) at 12,000 rpm for 3 min, sieved through an 80-mesh sieve to get homogeneous powder. The powder was mixed with ultrapure water at 1:15 (*w*/*v*), pH was adjusted to 9.0 with 1 M NaOH. After stirring at 300 rpm for 60 min under ambient conditions, the mixture was acidified to pH 4.5 with 1 M HCl, stood at 4 °C for 2 h. The precipitated protein fraction was recovered through centrifugal separation (8000 rpm, 15 min, 4 °C), redissolved in ultrapure water, and pH was reset to 8.0 with 1 M NaOH. Alkaline protease was added at 1% (*w*/*w*, enzyme/substrate), hydrolyzed in a thermostatic water bath at 50 °C, 250 rpm for 4 h. Enzymatic activity was terminated by thermal denaturation at 95 °C for 20 min, followed by rapid temperature reduction through immersion in an ice bath. The hydrolysate was vacuum-filtered with Whatman No. 1 paper (GE Healthcare, Chicago, IL, USA), then ultrafiltered (Model UF-100, Beijing Separation Tech. Co., Ltd., Beijing, China) with a 3 kDa PES membrane (0.3 MPa, 4 °C) to enrich small-molecule peptides (<3 kDa) with skin penetration potential. The permeate was concentrated by nanofiltration (Model NF-200, 200 Da polyamide membrane, 0.6 MPa, 4 °C), freeze-dried (−55 °C/10 Pa, 24 h) to get TPFP, which was stored in airtight containers at −20 °C for later use.

### 2.3. Peptide Sequence Identification

Peptide sequencing was performed by Sangon Biotech (Shanghai, China) through liquid chromatography-tandem mass spectrometry (LC-MS/MS) analysis. The sample was loaded onto an Easy-nLC 1200 system, separated on a C18 column (150 μm × 150 mm, 1.9 μm) with mobile phase A (0.1% formic acid in water) and B (20% 0.1% formic acid water-80% acetonitrile) at 600 nL/min, then analyzed by a Q-Exactive mass spectrometer (ESI positive mode, 300–1800 *m/z* scan range, resolution 70,000 at *m/z* 400). The collected raw MS files were de novo analyzed using PEAKS Studio 8.5 (Bioinformatics Solutions Inc., Waterloo, ON, Canada) and searched for the *P*. *suffruticosa* sequence database from NCBI. The precursor ion mass tolerance was set to 10 ppm.

### 2.4. Cell Culture

Human keratinocyte HaCaT cells were provided by HYcell biotechnology (Wuhan, China). Mouse skin melanoma B16F10 cells were obtained from Procell (Wuhan, China). HaCaT keratinocytes and B16F10 melanocytes were maintained in Dulbecco’s modified Eagle medium supplemented with 10% (*v*/*v*) fetal bovine serum and 1% (*v*/*v*) antibiotic solution (100 U/mL penicillin, 100 μg/mL streptomycin). Cell cultures were incubated at 37 °C in a humidified atmosphere of 5% CO_2_.

### 2.5. Determination of Cytotoxicity

To evaluate the in vitro safety of TPFP, the cytotoxicity of TPFP on different target cells was evaluated by CCK-8 assay as described previously [22]. In brief, target cells were plated onto 96-well microplates at 2 × 10^4^ cells per well and allowed to incubate at 37 °C for 24 h. Serially diluted TPFP (0, 20, 50, 100, and 200 μM), whose molar concentration was standardized by its average molecular weight (914.51 Da, identified via LC-MS/MS), was then introduced to the wells. Following a 24 h exposure period, cellular metabolic activity was assessed by adding 10 μL of CCK-8 reagent to each well. After approximately 3 h of additional incubation, absorbance at 450 nm was quantified spectrophotometrically using a microplate reader (DR-200Bs, Diatek, Wuxi, China).

### 2.6. UVB Irradiation and Peptide Treatment

UVB irradiation and peptide treatment were performed with modifications based on a previously reported method [15]. HaCaT keratinocytes were plated in six-well plates at a density of 5 × 10^5^ cells per well and subsequently organized into five distinct groups: a blank group (neither irradiation nor peptide exposure), a control group (exposed to UVB irradiation but without TPFP treatment), and three experimental groups treated with TPFP at different concentrations (20, 50, and 100 μM) prior to UVB irradiation. After washing with phosphate-buffered saline (PBS), the cells were subjected to UVB radiation at a dose of 30 mJ/cm^2^ using a PL-S 9W/01/2P UVB lamp (Philips, Amsterdam, The Netherlands), with a fixed distance of 10 cm between the light source and the target cells. The irradiance was measured using a UVX radiometer (UVP, Upland, CA, USA). Twenty-four hours after treatment, the cells and culture supernatants were collected for subsequent experiments.

### 2.7. ROS Production Measurement

After treatment with UVB irradiation (30 mJ/cm^2^) and different concentrations of TPFP, HaCaT cells were then incubated in the dark with cell-permeable fluorescent 2′,7′-dichlorofluorescin diacetate (DCFH-DA), which is an oxidation-sensitive fluorescence probe, for 30 min at 37 °C. Finally, treated cells were washed with ice-cold PBS three times and analyzed using a Nikon Eclipse C1 fluorescence microscope (Nikon, Tokyo, Japan).

### 2.8. Analysis of Intracellular SOD and CAT Activities

Treated cells (1 × 10^6^) were collected and homogenized on ice in 300 μL PBS, centrifuged at 10,000× *g* for 10 min at 4 °C. The supernatant was assayed for the intracellular activities of SOD and CAT using commercial kits, strictly adhering to manufacturer-provided protocols.

### 2.9. Determination of Pro-Collagen I, MMP-1 and HA Contents

To determine the effect of TPFP on the secretions of pro-collagen I, MMP-1 and HA of treated cells, the cellular supernatants were collected after 24 h of treatment and analyzed using ELISA kits (ELK Biotechnology, Wuhan, China). The contents of pro-collagen I, MMP-1 and HA were determined according to the manufacturer’s recommended protocols. The color change of the supernatant was measured spectrophotometrically at a wavelength of 450 nm, and the concentrations of pro-collagen I, MMP-1 and HA were calculated by comparing the OD of the samples to the standard curve.

### 2.10. Evaluation of Melanin Content

Melanin quantification was performed using an adapted version of a previously established spectrophotometric protocol [23]. In brief, B16F10 cells (2 × 10^4^ cells/well) were incubated in a 24-well plate for 24 h. The cells were exposed for 48 h with α-MSH (200 nM) to induce melanin synthesis in the presence or absence of TPFP. The cells without any treatment and treated only with α-MSH were used as the control and negative control, respectively. After the treatment, the cells were lysed in 1 M NaOH for 2 h at 60 °C with subsequent recovery of the pigment-containing supernatant by centrifugal separation. The total melanin contents were spectrophotometrically detected using a microplate reader at 405 nm. Experimental values were normalized and presented as a percentage relative to the melanin content of untreated control cultures.

### 2.11. Western Blot Analysis

Immunoblotting was employed to assess the expression levels of key melanogenic proteins, tyrosinase (TYR), tyrosinase-related protein 1 (TRP-1), and tyrosinase-related protein 2 (TRP-2), following a described protocol [24] with modifications. The B16F10 cells (4 × 10^5^) were seeded into 6-well plates and treated with α-MSH and TPFP (0, 20, 50, and 100 μM) for 48 h. Parallel cultures maintained without experimental intervention served as the reference control group. After being harvested, the cells were collected and completely lysed. Lysates were clarified by centrifugation (12,000 rpm, 5 min, 4 °C), and the supernatants were collected. Total protein content was quantified using the bicinchoninic acid method, and equal amounts of protein (40.0 μg) were mixed with a 5 × SDS-loading buffer and heated at 100 °C for 10 min and then subjected to a sodium dodecyl sulfate-polyacrylamide gel electrophoresis (SDS-PAGE) at 120 V for 90 min. Resolved proteins were electrophoretically transferred onto a polyvinylidene fluoride (PVDF) membrane. Membranes were blocked with 5% non-fat dried milk for 2 h, followed by overnight incubation with primary antibodies at 4 °C. After three 10 min washes with TBST, membranes were probed with horseradish peroxidase-conjugated secondary antibodies for 2 h. Finally, the Western blot bands were detected on X-ray films by an enhanced chemiluminescence (ECL) kit, and the AlphaEaseFC software, version 4.0.1 (Alpha Innotech, San Leandro, CA, USA) was used for the densitometric analysis of the obtained results. The intensities of individual bands were normalized to the intensity of the corresponding β-actin bands.

### 2.12. Statistical Analysis

All the mentioned tests were conducted with three independent biological replicates, with data presented as mean values with standard deviation (S.D.). Statistical computations were carried out using SPSS version 17.0 (IBM Corp., Armonk, NY, USA). Intergroup comparisons were analyzed by one-way analysis of variance with Tukey’s honest significant difference post hoc test. Statistical significance was defined at *p* ≤ 0.05.

## 3. Results

### 3.1. Sequence Characteristics of TPFP

To ensure the transparency and reproducibility of the sequencing data, a comprehensive dataset of total peony flower peptides has been compiled into a Supplementary Table S1. Through high-precision mass spectrometry sequencing, 54 unique peony flower-related peptides were identified, which mapped to 29 distinct proteins with no duplicate sequences—this uniqueness guarantees the reliability of the dataset for subsequent research. Notably, these peptides exhibited typical characteristics of small-molecule bioactive peptides, endowing them with great potential for biological application development.

In terms of structural features, the average amino acid length of the identified peptides is 8.2 residues, with 85.2% of them ranging from 5 to 10 amino acids. This length range is widely acknowledged to strike a balance between bioactivity retention and gastrointestinal absorption efficiency. Among all peptides, six-amino-acid variants are the most abundant (12 peptides, accounting for 22.2%), followed by seven-amino-acid (9 peptides, 16.7%) and eight-amino-acid (8 peptides, 14.8%) counterparts. Detailed information on the five most abundant peptides—including their sequences, molecular weights, relative contents, and protein sources—is summarized in Table 1. Regarding molecular mass, the average value of the TPFP is 914.51 Da, and 79.6% of the peptides fall within the 500–1100 Da range. This low molecular weight property allows the peptides to easily cross biological membranes (e.g., intestinal epithelial barriers) and be rapidly absorbed by organisms, laying a solid foundation for exerting potential physiological activities. The average molecular weight of 914.51 Da was used to standardize the molar concentrations of TPFP for all cell-based assays, ensuring reproducible dosing across experiments.

It should be noted that while the current study provides preliminary sequence and abundance information for TPFP, further research is warranted: precise in silico prediction of the bioactivities of individual peptides (especially the four high-abundance candidates) and subsequent in vitro/in vivo functional validation are essential to fully unravel their biological roles. In the present work, we first focused on evaluating the overall efficacy of the mixed TPFP, with the detailed characterization of single-peptide functions planned as a follow-up study.

### 3.2. Effect of TPFP on Cell Viability

**Figure 1:**
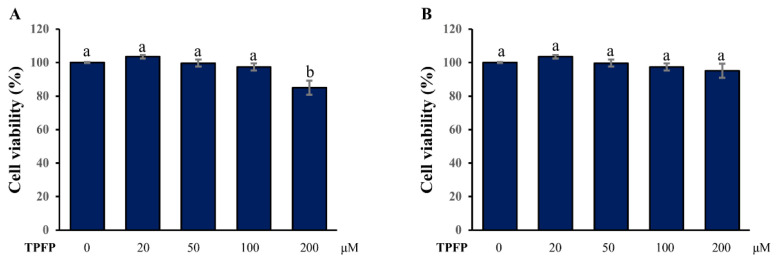
Effect of TPFP on the viability of HaCaT (**A**) and B16F10 (**B**) cells. Different letters above the bars represent statistically significant differences (one-way ANOVA, Tukey’s test, *p* < 0.05).

To evaluate the cytotoxicity of TPFP, HaCaT and B16F10 cells were treated with serial concentrations of TPFP for 24 h. As shown in Figure 1, compared with the untreated cells in the blank control, TPFP in the range of 20–100 μM is nontoxic to HaCaT cells, while 200 μM of TPFP could significantly reduce the cell viability to 85.12 ± 4.23% (*p* < 0.05). Meanwhile, TPFP in the range of 20–200 μM is nontoxic to B16F10 cells. Therefore, the concentrations of 20, 50 and 100 μM were used as low, medium and high dosages of TPFP in the subsequent experiments, respectively.

### 3.3. Inhibitory Effect of TPFP on Intracellularly Overproduced ROS

**Figure 2:**
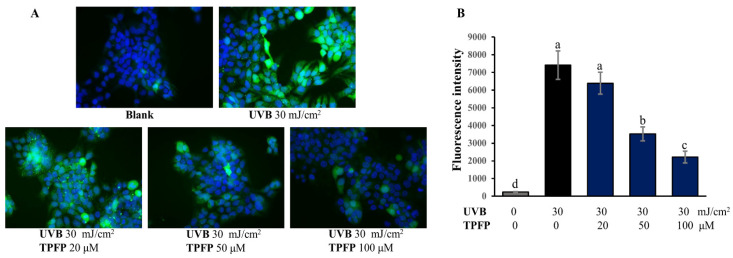
Effect of TPFP on intercellular ROS generation in UVB-irradiated HaCaT cells. (**A**) Representative image of intercellular ROS stained with DCFH-DA (original magnification ×200). (**B**) Bar graphs showing the fluorescence intensity of ROS levels. Different letters above the bars represent statistically significant differences (one-way ANOVA, Tukey’s test, *p* < 0.05).

The changes in intracellular ROS levels in the HaCaT cells were evaluated by measuring the intensity of DCFH fluorescence (Figure 2A). As Figure 2B shows, a significant increase in ROS level was observed in UVB-irradiated HaCaT cells. Treatment with TPFP (20, 50 and 100 μM) suppressed the overproduction of ROS in a dose-dependent manner. The 50 and 100 μM of TPFP significantly decreased intracellular ROS levels in UVB-irradiated HaCaT cells (*p* < 0.05). The results indicate a significant inhibitory effect of TPFP on the intracellular ROS level in UVB-irradiated HaCaT cells.

### 3.4. Regulatory Effect of TPFP on Antioxidant Enzyme Activities

**Figure 3:**
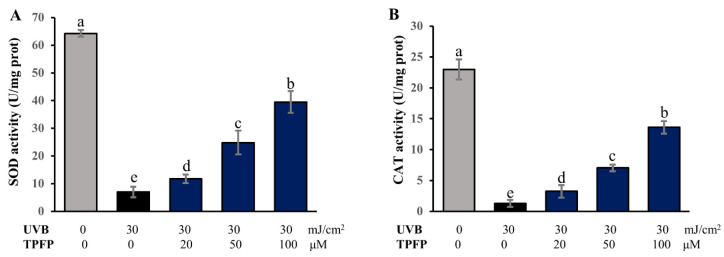
Regulatory effect of TPFP on SOD (**A**) and CAT (**B**) activities. Different letters above the bars represent statistically significant differences (one-way ANOVA, Tukey’s test, *p* < 0.05).

The antioxidant enzymes, such as superoxide dismutase (SOD) and catalase (CAT), are able to scavenge the overproduced ROS and protect cells from oxidative damage. However, after UVB irradiation, the activities of SOD and CAT were significantly decreased to 7.01 ± 1.94 and 1.28 ± 0.56 U/mg prot in oxidization-damaged cells (Figure 3). Treating with TPFP obviously enhanced SOD and CAT activities in a dose-dependent manner in the UVB-irradiated cells (*p* < 0.05). In the group treated with 100 μM of TPFP, the activities of SOD and CAT reached 39.50 ± 3.94 and 13.61 ± 1.04 U/mg prot, respectively. These results indicate that TPFP may inhibit damaging ROS accumulation through restoring antioxidant enzyme activities.

### 3.5. Effect of TPFP on Pro-Collagen I Degradation and MMP-1 Expression

**Figure 4:**
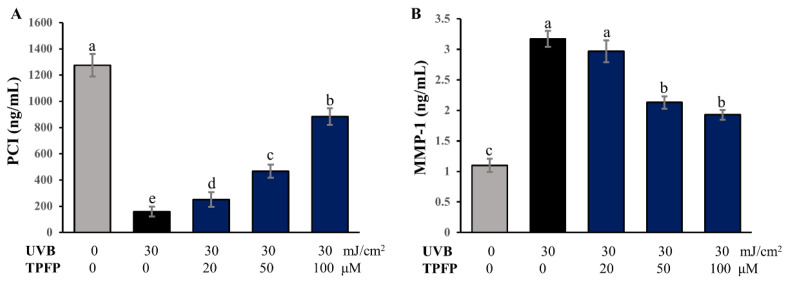
Anti-wrinkle effects of TPFP on pro-collagen I (**A**) and MMP-1 (**B**) contents in UVB-irradiated HaCaT cells. Different letters above the bars represent statistically significant differences (one-way ANOVA, Tukey’s test, *p* < 0.05).

The collagen secreted in normal adult skin is mainly type I collagen. The degradation of collagen leads to reduced skin elasticity and promotes skin wrinkling. After acute oxidative damage of skin cells, intracellular levels of pro-collagen I (PCI), the precursor of type I collagen, were significantly reduced (Figure 4A). However, different concentrations of TPFP had significant protective effects on the loss of PCI, which had been suppressed by UVB (*p* < 0.05). MMP-1 serves as the principal enzymatic mediator of type I collagen catabolism. As shown in Figure 4B, TPFP exhibited an inhibitory effect against UVB-induced MMP-1 expression, especially at concentrations of 50 and 100 μM. These observations implicate TPFP as a potential intervention against photoaging-associated wrinkle formation.

### 3.6. Protective Effect of TPFP on HA Content

**Figure 5:**
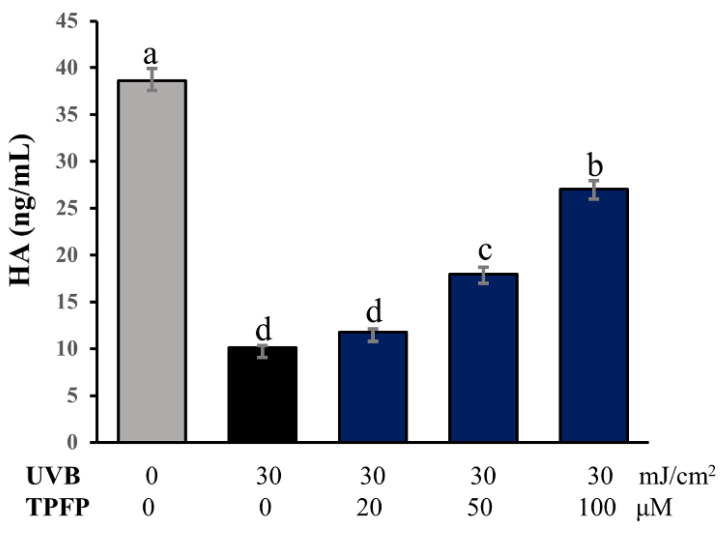
Effects of TPFP on hyaluronic acid (HA) content in UVB-irradiated HaCaT cells. Different letters above the bars represent statistically significant differences (one-way ANOVA, Tukey’s test, *p* < 0.05).

Hyaluronic acid (HA), a key molecule involved in skin moisture, has enormous potential to bind water, which helps the skin to retain moisture and maintain elasticity [25]. Therefore, the effect of TPFP on the HA content in UVB-irradiated HaCaT cells was investigated. As shown in Figure 5, compared with the control cells, the HA content was significantly decreased to 26.1% after UVB irradiation (*p* < 0.05). However, TPFP restored the decreased HA with increasing concentration, recovering skin moisturizing properties. Compared with the control cells, TPFP treatment at the concentration of 100 μM restored the HA content to 70.0% (*p* < 0.05).

### 3.7. Inhibitory Effect of TPFP on Melanin Contents

**Figure 6:**
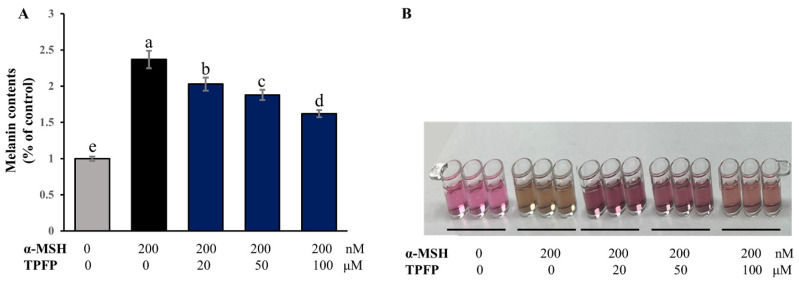
Inhibitory effect of TPFP on α-MSH-stimulated melanin production in B16F10 cells. (**A**) Bar graphs showing the melanin contents relative to control. (**B**) Representative image of melanin release in the culture medium. Different letters above the bars represent statistically significant differences (one-way ANOVA, Tukey’s test, *p* < 0.05).

After TPFP treatment for 48 h in the presence of α-MSH, a melanocyte-stimulating hormone, we explored the effects of TPFP on the melanogenesis response induced by α-MSH in B16F10 cells. As indicated in Figure 6, treatment with 200 nM of α-MSH alone markedly increased the level of melanin content to 237.1% of the control level (*p* < 0.05), while co-treatment with TPFP significantly decreased α-MSH-stimulated melanin production to 202.9%, 188.2% and 162.1%, respectively, in a concentration-dependent manner (*p* < 0.05).

### 3.8. Regulatory Effect of TPFP on the Expression of Melanogenesis-Related Enzymes

**Figure 7:**
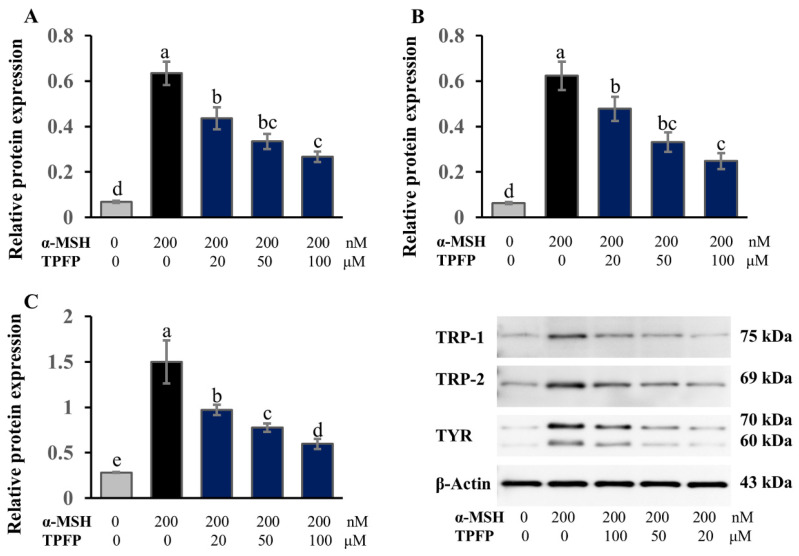
Inhibitory effect of TPFP on the protein-expression of melanogenic enzymes: TRP-1 (**A**), TRP-2 (**B**) and TYR (**C**). Different letters above the bars represent statistically significant differences (one-way ANOVA, Tukey’s test, *p* < 0.05). Protein expression levels were normalized to β-actin and quantified as relative fold change compared with the control group.

Melanin biosynthesis is enzymatically regulated by a tripartite system comprising TYR as the rate-limiting enzyme, supported by TRP-1 and TRP-2 [26]. Therefore, we investigated the effect of TPFP on the protein-expression levels of TRP-1, TRP-2 and TYR (Figure 7) in α-MSH-stimulated B16F10 cells. Our results revealed that all three enzymes were significantly induced by α-MSH stimulation, while the application of 20 to 100 μM TPFP dose-dependently suppressed the production of these melanogenesis-related enzymes (*p* < 0.05). These results collectively indicated that TPFP has an inhibitory effect on melanin synthesis through decreasing the protein-expression levels of TRP-1, TRP-2 and TYR.

## 4. Discussion

In this study, we systematically explored the potential skin-health-promoting effects of TPFP by evaluating its antioxidant, anti-wrinkle, moisturizing, and whitening activities in HaCaT keratinocytes and B16F10 melanoma cells. Prior to functional assessments, we first characterized the sequence properties of TPFP and verified its cytotoxicity on target cells to ensure the safety of subsequent experiments. The results revealed that TPFP is composed of 54 unique small-molecule peptides, with an average amino acid length of 8.2 residues and an average molecular weight of 914.51 Da. Among these, 85.2% of the peptides fall within the 5–10 amino acid range and 79.6% within the 500–1100 Da molecular weight range. This structural feature aligns with the optimal peptide size (400–600 Da) highlighted in anti-melanogenesis research, where smaller peptides exhibit higher metal-chelating and enzyme-inhibitory activities. For instance, fish-scale-gelatin-derived peptides (<667 Da) and Colla corii asini-derived tetrapeptides (<500 Da) have been shown to strongly inhibit tyrosinase by minimizing steric hindrance at the enzyme’s active site [27,28]. Cytotoxicity assays confirmed that TPFP (20–100 μM) exhibited no significant toxicity to HaCaT and B16F10 cells, providing a safe concentration window for subsequent functional evaluations. This in vitro cytotoxicity evaluation preliminarily verifies the biocompatibility of TPFP, which is consistent with the non-toxicity of tree peony seed protein hydrolysates to HepG2 cells [12], suggesting Paeonia-derived bioactive peptides have inherent safety characteristics. Moreover, TPFP is composed of small-molecule peptides with 5–10 amino acids and a molecular weight of 500–1100 Da, with moderate hydrophobic amino acid content, which is the structural basis for its non-toxicity. However, this preliminary in vitro safety study is limited to two cell lines, and more scientific and systematic toxicological studies (e.g., in vivo animal model tests, acute and long-term toxicity evaluations) are needed in subsequent research to further validate the safety of TPFP.

Oxidative stress induced by UVB irradiation is a primary driver of skin photoaging, characterized by excessive reactive oxygen species (ROS) production and impaired antioxidant enzyme activity [29]. Consistent with this mechanism, our results showed that UVB irradiation significantly elevated intracellular ROS levels in HaCaT cells while reducing superoxide dismutase (SOD) and catalase (CAT) activities—key enzymes responsible for ROS scavenging. TPFP treatment dose-dependently inhibited UVB-induced ROS overproduction (with 50 and 100 μM exerting significant effects) and restored SOD/CAT activities to near-normal levels. This dual antioxidant mechanism—direct ROS scavenging and enhancement of endogenous antioxidant defenses—echoes the multifunctional role of bioactive peptides. For example, antioxidant peptides derived from walnut protein exhibited good antioxidant properties, protected HT22 cells against oxidative damage, inhibited ROS generation, and enhanced the activities of antioxidant enzymes, including catalase (CAT), superoxide dismutase (SOD), and glutathione peroxidase (GSH-Px) [30].

Skin wrinkle formation and moisture loss are closely linked to collagen degradation, matrix metalloproteinase (MMP) activation, and reduced hyaluronic acid (HA) content [31]. UVB irradiation typically decreases pro-collagen I (PCI, precursor of type I collagen) and upregulates MMP-1 (a key collagen-degrading enzyme), while reducing HA—a critical moisturizing molecule. TPFP treatment reversed PCI loss, inhibited MMP-1 overexpression, and restored HA content to 70% of the normal level at 100 μM. These observations align with the anti-aging mechanism of plant peptides described by Aguilar-Toalá et al. [10], where small-molecule peptides from canary seed interact with skin-aging enzymes (e.g., MMP-1) via hydrophilic residues and penetrate lipid bilayers via hydrophobic regions, enhancing bioavailability. Notably, TPFP-mediated PCI restoration exhibited a distinct dose-dependent pattern, while its inhibitory effect on MMP-1 did not follow this proportional trend. This apparent discrepancy can be ascribed to the involvement of distinct intracellular signaling pathways in the regulation of these two molecules: PCI synthesis is primarily governed by the TGF-β/Smad pathway, whereas MMP-1 expression is predominantly controlled by the MAPK/AP-1 signaling cascade [32].

Hyperpigmentation, driven by α-melanocyte-stimulating hormone (α-MSH)-induced melanin overproduction, is mediated by tyrosinase (TYR), tyrosinase-related protein 1 (TRP-1), and TRP-2 [33]. Our results showed that TPFP dose-dependently suppressed α-MSH-induced melanin synthesis and downregulated TYR/TRP-1/TRP-2 expression. This whitening mechanism is consistent with the peptide-TYR interaction model proposed by Kose et al. [34], where peptides derived from *Spirulina platensis* phycocyanin inhibit TYR by binding to its active site (via hydrogen bonds and hydrophobic interactions) or chelating copper ions. Further, Wang et al. [35] demonstrated that peptides from *Atrina pectinata* mantle interact with TYR’s critical residues (e.g., His263, Val283) to stabilize the peptide-enzyme complex.

Notably, TPFP’s multifunctional efficacy (antioxidant, anti-wrinkle, moisturizing, whitening) with high biocompatibility addresses the limitations of single-target skin care ingredients. Conventional whitening agents (e.g., arbutin) often suffer from poor stability and limited efficacy [36], while TPFP’s peptide mixture leverages structural diversity to target multiple skin damage pathways. This aligns with the trend toward natural, multifunctional peptides in cosmeceuticals, where plant-derived hydrolysates offer a sustainable alternative to synthetic compounds. For example, tyrosinase inhibitory peptides from enzyme-hydrolyzed royal jelly have been shown to exhibit strong binding affinity to TYR via hydrophobic and hydrogen bonding interactions [37], supporting the notion that mixed peptide fractions enhance efficacy through synergistic mechanisms.

Despite these promising results, this study has several limitations. First, the specific peptides responsible for individual functions (e.g., antioxidant vs. whitening) within TPFP remain uncharacterized. Future research should isolate and purify key peptides, combined with in silico docking (e.g., targeting TYR or MMP-1) to clarify their structure–activity relationships. Second, the in vivo efficacy of TPFP requires verification in animal models or clinical trials, as in vitro results may not fully reflect human skin physiology. Third, the impact of peptide cyclization or modification (e.g., methylation) on TPFP’s stability and efficacy was not explored; such strategies have been shown to enhance peptide skin penetration and metabolic stability [38] and could further optimize TPFP’s performance.

## 5. Conclusions

This study identified 54 unique small-molecule peptides in tree peony flower protein hydrolysate (TPFP) and systematically evaluated its skin-health-promoting potentials. Functional assays demonstrated that TPFP exhibits comprehensive benefits, including antioxidant activity by inhibiting UVB-induced reactive oxygen species (ROS) overproduction and restoring SOD/CAT activities, anti-wrinkle and moisturizing effects by protecting pro-collagen I, inhibiting MMP-1, and recovering hyaluronic acid (HA) content in HaCaT cells, as well as whitening effects by suppressing α-MSH-induced melanin synthesis through downregulating TYR, TRP-1, and TRP-2 expression in B16F10 cells. However, limitations exist: the structural characteristics of individual peptides remain unanalyzed, and their specific functional mechanisms and in vivo efficacy lack verification. Future research should focus on analyzing peptide structures, isolating key functional components, elucidating molecular mechanisms, and conducting animal and clinical trials to optimize extraction and purification processes, thereby promoting TPFP’s industrial application in cosmetics and functional foods.

## Figures and Tables

**Table 1:** Top 5 abundant peptides in TPFP.

Peptide Sequence	Length (AA)	Mass (Da)	Protein Accession	Protein Name	Relative Abundant (%)
AIAQDTL	7	730.39	UUA01456.1	NADH-plastoquinone oxidoreductase subunit 2 (chloroplast)	17.74
LRQGGPPA	8	794.44	WGJ63705.1	DELLA3 protein	14.58
LTFSVFPS	8	896.46	ANC98505.1	beta-tubulin, partial	10.66
RVTPQPGVPPEE	12	1304.67	UZP82119.1	ribulose-1,5-bisphosphate carboxylase/oxygenase large subunit	5.19
TEAPLNPK	8	868.47	AEK70331.1	Actin	4.45

## Data Availability

The data presented in this study are included in the article, and the detailed peptide sequence information is available in Supplementary Table S1. Further inquiries can be directed to the corresponding author.

## Supplementary Materials

The following supporting information can be downloaded at: https://www.mdpi.com/article/10.3390/antiox15030350/s1, Table S1: Identified Peptides from TPFP.
