# Supplementary material for: Natural Bioactive Peptides from Tree Peony Flowers: Multifunctional Effects on Skin Antioxidation, Wrinkle Reduction, Moisturization, and Melanin Inhibition
Source: Antioxidants (Basel). 2026 Mar 11;15(3):350. doi: 10.3390/antiox15030350 (PMC13023428; doi:10.3390/antiox15030350)
Supplement: Supplementary file 1 [file antioxidants-15-00350-s001.zip › antioxidants-4196462-supplementary.pdf]

Supplementary Table S1: Identified Peptides from TPFP

No.	Peptide Sequence	Amino Acid Length	Molecular Weight (Da)	Protein Accession No.	Protein Name	Relative Abundance (%)
1	L.TEAPLNPK.A	8	868.4654	AEK70331.1	Actin	4.45
2	K.VVAPPERKY.S	9	1057.5920	AEK70331.1	Actin	1.93
3	G.FAGDDAPR.A	8	847.3824	AEK70331.1	Actin	0.40
4	E.SGPSIVH.R	7	695.3602	AEK70331.1	Actin	0.91
5	A.GFAGDDAPR.A	9	904.4039	AEK70331.1	Actin	0.12
6	R.VAPEEHPVLLTEAPLNPK.A	18	1953.0570	AEK70331.1	Actin	0.51
7	T.EAPLNPK.A	7	767.4177	AEK70331.1	Actin	0.42
8	K.AGFAGDDAPR.A	10	975.4410	AEK70331.1	Actin	0.83
9	E.EHPVLL.T	6	706.4014	AEK70331.1	Actin	3.87
10	A.GFAGDDAPRA.V	10	975.4410	AEK70331.1	Actin	0.24
11	R.LDLAGR.D	6	643.3653	AEK70331.1	Actin	1.48
12	K.AGFAGDDAPRA.V	11	1046.4780	AEK70331.1	Actin	0.65
13	K.SSSSVEKN(+.98)YELPDGQ.V	15	1639.7213	AEK70331.1	Actin	0.00
14	E.SSDTIDNVKAK.I	11	1176.5986	AER12038.1	Ubiquitin extension protein	0.63
15	S.DTIDNVKAK.I	9	1002.5345	AER12038.1	Ubiquitin extension protein	1.05
16	K.Q(-17.03)LEDGRTL.A	8	913.4505	AER12038.1	Ubiquitin extension protein	1.00
17	E.SSDTIDNVK.A	9	977.4666	AER12038.1	Ubiquitin extension protein	0.20
18	F.RVTPQPGVPPEE.A	12	1304.6724	UZP82119.1	ribulose-1,5-bisphosphate carbox-ylase/oxygenase large subunit	5.19

19	Y.YTPDYKTK.D	8	1014.5022	UZP82119.1	ribulose-1,5-bisphosphate carbox-ylase/oxygenase large subunit	0.25
20	Y.YTPDYK.T	6	785.3596	UZP82119.1	ribulose-1,5-bisphosphate carbox-ylase/oxygenase large subunit	0.32
21	M.RIINEPT.A	7	841.4658	AFA51946.1	Heat shock protein 70	2.70
22	M.RIINEPTAA.A	9	983.5400	AFA51946.1	Heat shock protein 70	1.07
23	R.IINEPTAAAIAYGLDK.K	16	1658.8879	AFA51946.1	Heat shock protein 70	0.18
24	E.IIAN(+.98)DQGNRTTPS.Y	13	1386.6740	AFA51946.1	Heat shock protein 70	0.14
25	K.RLIGR.R	5	613.4023	AFA51946.1	Heat shock protein 70	0.19
26	E.TSVPTPPKKE.V	10	1082.5972	AJI44066.1	dihydrolipoyllysine-residue acetyltransferase	0.13
27	E.VVEEPVSSPEPK.V	12	1295.6609	AJI44066.1	dihydrolipoyllysine-residue acetyltransferase	0.66
28	L.VADDEWLK.G	8	974.4709	AEE60889.1	malate dehydrogenase	0.68
29	M.NHPGQIGN(+.98)GY.A	10	1056.4624	ABW74483.1	elongation factor	0.36
30	S.VFPSPK.V	6	673.3799	ANC98505.1	beta-tubulin, partial	1.54
31	M.LTFSVFPS.P	8	896.4644	ANC98505.1	beta-tubulin, partial	10.66
32	T.FLVPR.D	5	630.3853	QCG69902.1	RNA polymerase	1.39

					beta subunit	
33	G.DKPVSVF.G	7	790.4225	ANC98507.1	glyceraldehyde 3-phosphate dehydrogenase, partial	1.10
34	F.RVPTVD.V	6	685.3759	ANC98507.1	glyceraldehyde 3-phosphate dehydrogenase, partial	3.40
35	K.IERPLF.Q	6	773.4435	ADB28916.1	chalcone synthase	1.84
36	L.IEDVWK.T	6	788.4069	WDZ04376.1	anthocyanin 5-O-glucosyltransferase	1.45
37	P.RNPVHI.M	6	734.4188	UUA01031.1	RNA polymerase beta subunit	0.00
38	C.TVIDAPGHR.D	9	964.5090	ANC98511.1	elongation factor 1 alpha, partial	0.15
39	M.A(+42.01)TIIAPH.Q	7	763.4229	QFG70406.1	annexin	2.05
40	R.DYSRWPDKPE.G	10	1291.5833	AEN71544.1	flavanone 3-hydroxylase	0.21
41	V.STDLIHW.L	7	870.4236	UHU35850.1	vacuolar invertase 2	0.48
42	T.DLIHW.L	5	682.3439	UHU35850.1	vacuolar invertase 2	0.20
43	R.VATVSLPR.S	8	841.5021	|TRYP_PIG	Trypsin	0.23
44	E.RVVDTPITE.A	9	1028.5502	AKA20327.1	pyruvate dehydrogenase	0.62
45	L.KLSVPT.L	6	643.3904	AVY53540.1	diacylglycerol acyltransferase	0.30
46	L.SQPFF.V	5	624.2907	AAK72818.1	ATP synthase beta subunit, partial (chloroplast)	0.00
47	A.LRQGGPPA.F	8	794.4398	WGJ63705.1	DELLA3 protein	14.58

48	G.ATPFF.T	5	581.2849	QMQ92488.1	photosystem I P700 chlorophyll A apoprotein A1	0.37
49	K.WGNLPK.K	6	713.3860	QIG55725.1	MYB transcription factor	0.13
50	F.KASSSK.A	6	606.3337	QIG55697.1	MYB transcription factor	0.32
51	I.AIAQDTL.F	7	730.3861	UUA01456.1	NADH-plastoquinone oxidoreductase subunit 2 (chloroplast)	17.74
52	L.TVTVPK.D	6	643.3904	UQK84915.1	HSP17.8	1.72
53	S.GILN(+.98)PAG.P	7	641.3384	AFI61909.1	ethylene insensitive 3-like 3 protein	0.40
54	E.EDLIIIK.A	7	842.5113	QIG55688.1	MYB transcription factor	0.22

Table Notes: **1.** Molecular weights were calculated based on the peptide sequences; **2.** Relative abundances were determined by normalization of LC-MS/MS signal intensities; **3.** Protein accession numbers refer to the NCBI protein database (updated March 2025).
